# Design and performance of a sericin-alginate interpenetrating network hydrogel for cell and drug delivery

**DOI:** 10.1038/srep12374

**Published:** 2015-07-24

**Authors:** Yeshun Zhang, Jia Liu, Lei Huang, Zheng Wang, Lin Wang

**Affiliations:** 1 Research Center for Tissue Engineering and Regenerative Medicine, Union Hospital, Huazhong University of Science and Technology, Wuhan, Hubei, China 430022; 2 Department of Surgery, Union Hospital, Huazhong University of Science and Technology, Wuhan, Hubei, China 430022; 3 Department of Clinical Laboratory, Union Hospital, Huazhong University of Science and Technology, Wuhan, Hubei, China 430022; 4 Medical Research Center, Union Hospital, Huazhong University of Science and Technology, Wuhan, Hubei, China 430022

## Abstract

Although alginate hydrogels have been extensively studied for tissue engineering applications, their utilization is limited by poor mechanical strength, rapid drug release, and a lack of cell adhesive ability. Aiming to improve these properties, we employ the interpenetrating hydrogel design rationale. Using alginate and sericin (a natural protein with many unique properties and a major component of silkworm silk), we develop an interpenetrating polymer network (IPN) hydrogel comprising interwoven sericin and alginate double networks. By adjusting the sericin-to-alginate ratios, IPNs’ mechanical strength can be adjusted to meet stiffness requirements for various tissue repairs. The IPNs with high sericin content show increased stability during degradation, avoiding pure alginate’s early collapse. These IPNs have high swelling ratios, benefiting various applications such as drug delivery. The IPNs sustain controlled drug release with the adjustable rates. Furthermore, these IPNs are adhesive to cells, supporting cell proliferation, long-term survival and migration. Notably, the IPNs inherit sericin’s photoluminescent property, enabling bioimaging *in vivo*. Together, our study indicates that the sericin-alginate IPN hydrogels may serve as a versatile platform for delivering cells and drugs, and suggests that sericin may be a building block broadly applicable for generating IPN networks with other biomaterials for diverse tissue engineering applications.

Hydrogels, three-dimensional polymeric networks capable of swelling after absorbing large amount of water^1^, have been widely used in tissue engineering and regenerative medicine^2^. Owing to their biocompatibility and biodegradability, natural polymers are extensively studied for hydrogel fabrication. Among them, alginate, a polysaccharide produced by brown algae, has been used in numerous biomedical applications including tissue repair and drug delivery^3^. However, an ionically-crosslinked alginate hydrogel has poor mechanical strength making it difficult to engineer a hydrogel with defined geometrics^4^ and often causing failure in load bearing capability, especially in an environment containing monovalent ions^5^. Although oxidation and covalent crosslinking can improve the mechanical strength of an alginate hydrogel, these chemical modifications compromise other alginate properties and reduce its biocompatibility^6^. Moreover, alginate hydrogels are not cell-adhesive as alginate is relatively inert in interacting with integrins of mammalian cells^7^. Although alginate’s cell adhesive capability can be improved through covalently bound with specific adhesion motifs such as RGD peptide, YIGSR peptide^8^,^9^, or IKVAV peptide^10^, costly peptide synthesis and cell-type specific requirements for these adhesive peptides limit their applications.

A possible alternative to improve the properties of alginate hydrogels is to generate an interpenetrating network (IPN) hydrogel of alginate with other polymers. IPN is a combination of polymers in a network form. At least one polymer is synthesized and/or crosslinked in the presence of the other, either simultaneously or sequentially^11^. The combination of favorable properties of each constituent polymer results in a new hybrid system with the properties that are often significantly improved or substantially different from those of the individual polymers^12^. Therefore, designing and fabricating an alginate IPN hydrogel with other materials is a feasible approach to obtain improved mechanical properties, bio-adhesion, and drug release kinetics^12^.

Sericin, a major component of silk, glues fibroin (another component of silk) together to form robust cocoons. Sericin consists of 17–18 amino acids with ample amount of polar side chains made of hydroxyl, carboxyl and amino groups that can be used for crosslinking^13^,^14^. Sericin was reported to have diverse biological activities, such as anti-oxidation, anti-bacteria, anti-coagulation and promoting cell growth and differentiation^15^,^16^,^17^,^18^,^19^. In the field of regenerative medicine, owing to its hydrophilic and biodegradable characteristics, sericin is mostly copolymerized or blended with other polymers to form various scaffolds in order to help obtain improved properties for biomedical applications^20^,^21^,^22^,^23^,^24^,^25^. Although sericin reportedly formed several semi-IPNs with other polymers including polyacrylamide and ploy(aspartic acid)^26^,^27^,^28^ where sericin was often blended with a crosslinked polymer network^26^,^27^,^28^, the use of sericin with biopolymers to generate a full IPN hydrogel with double networks has never been explored. That is because in their studies sericin is difficult to be crosslinked on its own as it was largely degraded due to the conventional extraction process (involving high heat and alkaline), thus becoming amorphous with low molecular weights^29^. Previously, we overcame this difficulty by extracting sericin with a well-preserved protein profile from a fibroin-deficient mutant *Bombyx mori* silkworm. Using this type of sericin, we generated a novel crosslinked pure sericin 3D hydrogel^13^. This hydrogel is highly biocompatible, naturally cell-adhesive, elastic and photoluminescent. The generation of such a pure sericin hydrogel enables the further investigation of fabricating the IPN hydrogels of sericin with other polymers.

In this study, we hypothesize that an IPN hydrogel prepared from alginate and sericin would help improve alginate hydrogel’s properties in four ways: (1) enhancing mechanical strength; (2) improving degradation kinetics; (3) allowing *in vivo* tracking by photoluminescence; (4) enabling cell adhesion. Using calcium ion and glutaraldehyde as the crosslinking agents for alginate and sericin, respectively, we have successfully generated an IPN hydrogel with interwoven sericin and alginate double networks. This hydrogel exhibits improved mechanical strength and has more stable degradation kinetics when compared to alginate hydrogels. The IPN hydrogel shows the excellent cell-adhesive property and effectively supports the proliferation and migration of mouse myoblasts. Moreover, it releases drugs in a sustained manner. Thus, this sericin-alginate IPN hydrogel may serve as a versatile platform for cell or drug delivery. Further, this work suggests that sericin may be broadly applicable to generate IPN networks with other synthetic or natural biomaterials for improved properties for tissue engineering applications.

## Results and Discussion

### Synthesis of the sericin-alginate IPN hydrogels

**Figure 1:**
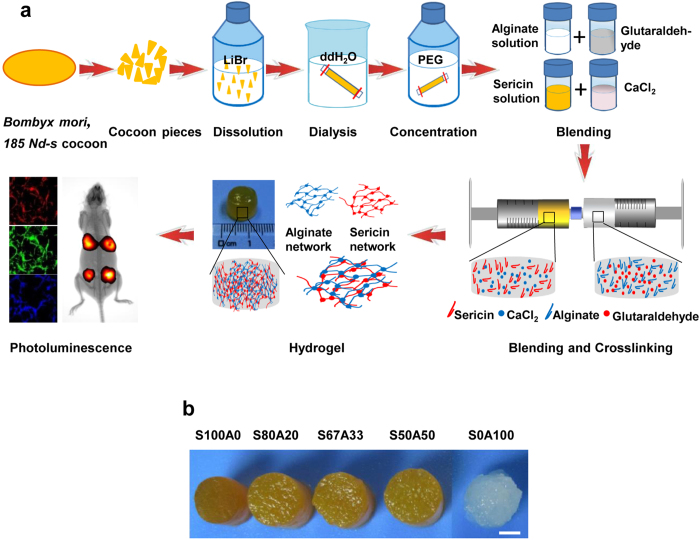
Schematics describing a strategy of fabricating the sericin-alginate hydrogels with double interpenetrating networks and the images of these IPN hydrogel samples. (**a**) A flow chart shows the procedure of sericin extraction, and the fabrication and bioimaging of an IPN hydrogel with the double networks. (**b**) Photographs of the sericin-alginate IPN hydrogels fabricated using different sericin-to-alginate ratios (v/v). Scale bar, 5 mm.

The double network sericin-alginate hydrogels were fabricated by mixing the alginate solution containing glutaraldehyde in a syringe and the sericin solution containing CaCl_2_ in another syringe via injection (see “Methods” for details; Fig. 1a). After mixing, the solution was incubated at room temperature to obtain the sericin-alginate IPN hydrogels. The five different ratios (v/v) of sericin (S) to alginate (A) were tested, 1:0, 4:1, 2:1, 1:1, and 0:1 (See “Methods” for details). The resulting IPN hydrogels were termed accordingly, S100A0, S80A20, S67A33, S50A50, and S0A100 (Fig. 1b). The IPN hydrogels exhibited yellowish appearance, which was due to the yellowish color of silk produced by the mutant silkworm (*Bombyx mori, 185 Nd-s*) we used.

### Morphology of the IPN hydrogels

**Figure 2:**
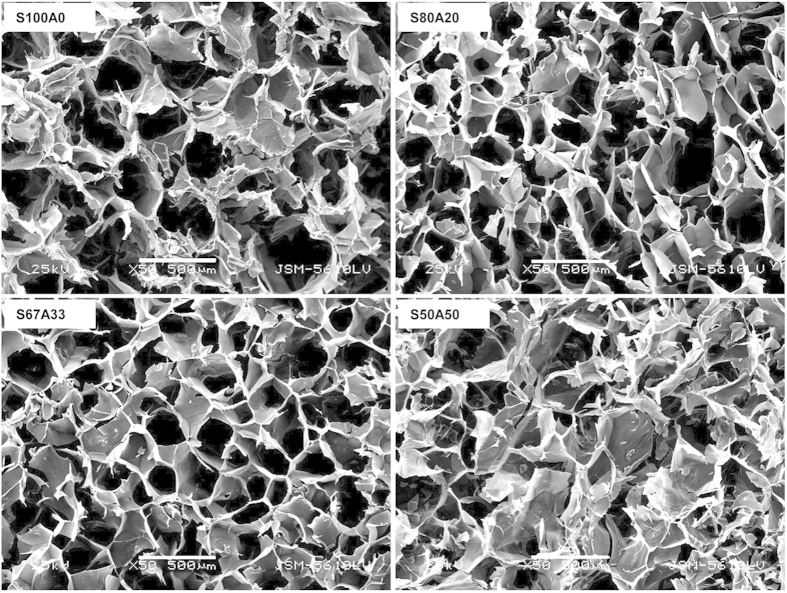
The porous microstructure of the sericin-alginate IPN hydrogels. The micrographs obtained using SEM show the random regions in the lyophilized samples of the pure sericin hydrogel (S100A0) and the IPN hydrogels (S80A20, S67A33 and S50A50) frozen at −80^o^C. Scale bars, 500 μm.

Porosity is tightly correlated with mechanical performance of a matrix as it affects encapsulation of biochemical agents, supply of nutrients and oxygen, and removal of waste products^30^. The lyophilized sericin-alginate IPN hydrogels had a highly porous structure (Fig. 2). The pore diameters of S100A0 (138.66 μm), S80A20 (105.23 μm), S67A33 (98.57 μm), and S50A50 (79.82 μm) were reduced as the sericin-to-alginate ratios within the IPN hydrogels decreased (Fig. 2; Table 1). However, regardless of the sericin-to-alginate ratios, all the IPN hydrogels had high porosity, approximately 90%, similar to the pure sericin hydrogel (Table 1). These results indicate that the sericin-alginate IPN hydrogels are highly porous, which would favor effective nutrient or gas exchange for cells^31^.

### Mechanical property

**Figure 3:**
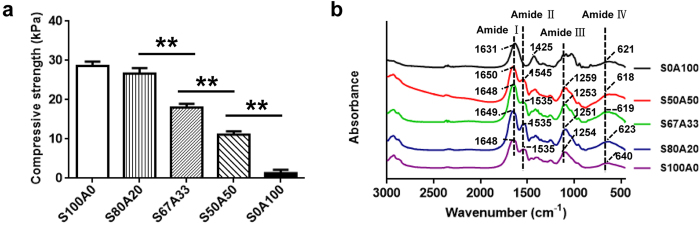
The mechanical strength and the FTIR spectra of the IPN hydrogels. (**a**) Quantification of compressive moduli of the pure sericin (S100A0), the three sericin/alginate IPN hydrogels (S80A20, S67A33, and S50A50), and the pure alginate hydrogel (S0A100) (n = 3 per group; ANOVA test; **P < 0.01). (**b**) FTIR spectra show the absorption peaks of amide I, II, III and IV (indicated) of the pure sericin (S100A0, purple line), the three sericin/alginate IPN hydrogels (S80A20, blue line; S67A33, green line; and S50A50, red line), and the pure alginate hydrogel (S0A100, black line).

We next examined the mechanical property of these IPN hydrogels. The alginate hydrogel had low mechanical strength as its compressive modulus was approximately 1 kPa, whereas the modulus of the pure sericin hydrogel was considerably higher, reaching 28 kPa (Fig. 3a). The compressive modulus of these IPN hydrogels increased as the alginate content decreased (*P* < 0.01) (Fig. 3a), suggesting that by adjusting the formulations of sericin and alginate, the mechanical strength of the IPN hydrogels can be flexibly regulated. Such mechanical tunability would provide handling convenience and help to meet a range of mechanical strength required for repairing different tissues, such as neuronal tissue^32^, myocardium^33^, and skeletal muscle^32^.

### Secondary structural conformations of the IPN hydrogels

The fourier transformation infrared spectroscopy (FTIR) is used to evaluate the structure of proteins and polypeptides^34^. The structure of protein is usually interpreted in terms of its absorption bands that are based on the vibration of a structural repeat. Among nine characteristic absorption bands (amides A, B and I-VII), the amide I (1690-1600 cm^−1^) primarily arising from the C=O stretching vibration is useful for revealing sericin protein secondary structures^35^. The amide I peaks of the IPN S80A20 (1649 cm^−1^), S67A33 (1648 cm^−1^) and S50A50 (1650 cm^−1^) were similar to that of S100A0 (1648 cm^−1^) (Fig. 3b), suggesting that the sericin networks within these IPNs do not have major structural transformations in comparison to the pure sericin hydrogel.

### Swelling behavior

**Figure 4:**
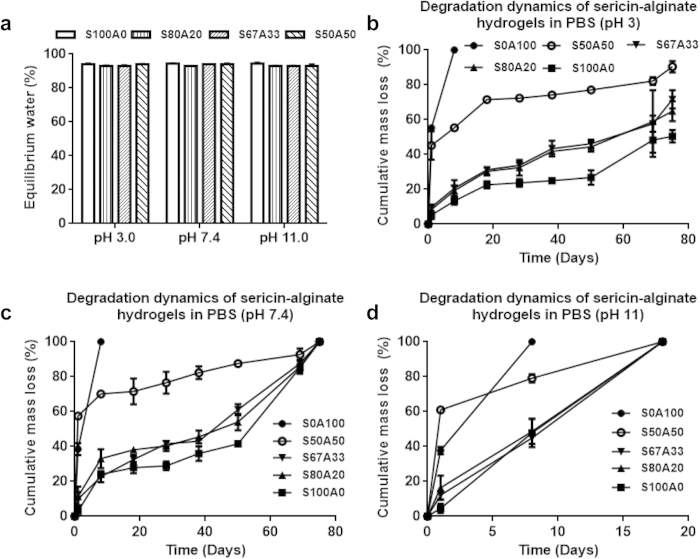
The swelling behavior and degradation kinetics of the sericin-alginate IPN hydrogels. (**a**) The equilibrium water content of the IPN hydrogels 24 hours after immersion into PBS with different pH (3, 7.4, 11) at 37 °C (n = 3 per group). (b, c, d) The cumulative weight loss of the IPN hydrogels over time in PBS with different pH (3, 7.4, 11) at 37 °C (n = 3 per group per time point).

Swelling behavior is an important property of hydrogels as many of their applications rely on their water absorbing capability. We next tested the swelling behavior of the IPN hydrogels at three different pH conditions (3, 7.4 and 11). The alginate hydrogel disassembled rapidly and nearly collapsed within 24 hours at all pH conditions tested. In contrast, at all pH conditions tested, the equilibrium water content of the IPN hydrogels and that of the pure sericin hydrogel (S100A0) were close, approximately 94% (Fig. 4a), indicating a similar capacity for water absorption regardless of IPN composition and pH conditions. This is likely due to the high abundance of hydrophilic groups in both sericin and alginate.

### Degradation kinetics

Hydrogel stability is important because it is directly related to the gel performance. Ionically-crosslinked alginate hydrogels were not stable in physiological environment^36^ and disintegrated rapidly in the presence of calcium chelators (e.g., phosphates) or monovalent ions (e.g., Na^+^ or K^+^)^36^,^37^. In contrast, the pure sericin hydrogel was relatively stable while degrading. At the acid and neutral conditions (pH 3, 5, 7.4), the pure sericin hydrogel took over 53 days to degrade^13^. Thus, the presence of a sericin network would presumably improve the stability of the sericin-alginate IPN hydrogels. Indeed, compared to the alginate hydrogel (S0A100), the degradation of three types of IPNs was significantly improved at all the pH conditions tested (Fig. 4b,c,d). Consistent with the degradation features of pure sericin hydrogels reported previously^13^, all the IPNs degraded faster (less than 20 days) in the alkaline (pH 11) condition than in the acidic or neutral conditions (up to 75 days). This may be because sericin has the isoelectric point approximately 3.8 and contains the higher content of acidic amino acids than that of alkaline amino acids^13^. Of note, while the degradation behavior of the two IPNs, S80A20 and S67A33, was similar to that of the pure sericin hydrogel (S100A0) at all the tested pH (Fig. 4b,c,d), the IPN S50A50′s degradation was distinctly different from the others, much faster (Fig. 4b,c,d), suggesting that high alginate content can significantly reduce the stability of the IPNs, reflecting the crucial role of sericin in stabilizing the IPNs.

### Photoluminescent property

**Figure 5:**
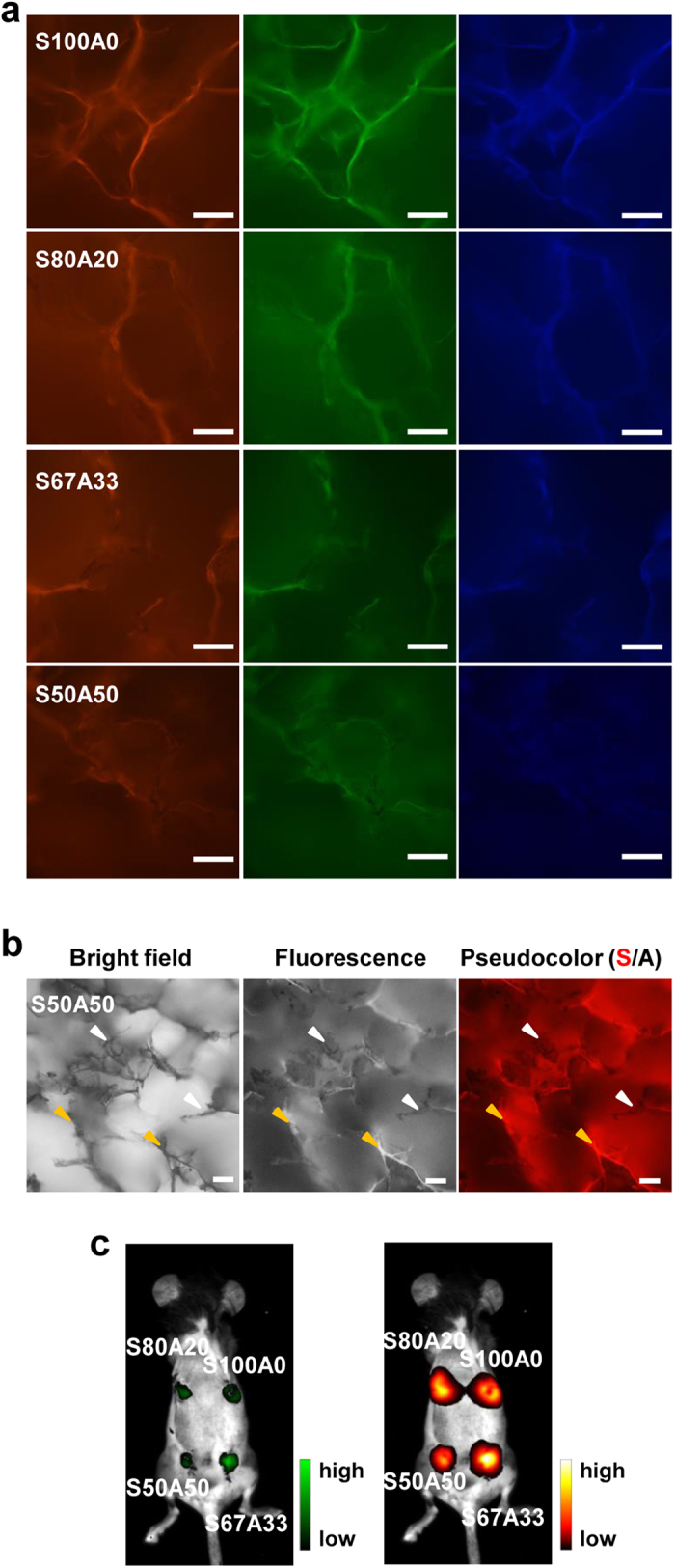
The photoluminescent property of the sericin-alginate IPN hydrogels. (**a**) 3D micro-structures of the IPNs were observed using confocal laser scanning microscopy under the light at the different wavelengths (left, imaged with the excitation wavelength 530-550 nm with the filter allowing the passage of the emission wavelength 575–625 nm; middle, imaged with 460–495 nm and with the filter allowing the passage of the emission wavelength 510 nm; right, imaged with the excitation wavelength 360–370 nm and with the filter allowing the passage of the emission wavelength >420 nm). Scale bars, 100 μm. (**b**) The double interpenetration networks within the IPN hydrogel (S50A50) were black when observed under the bright field (left). The fluorescent (middle) and corresponding pseudocolored (right) images of this IPN hydrogel show the fluorescent ridges (yellow arrowheads) of the sericin network and the black, non-fluorescent structure of the alginate network (white arrowheads). Scale bars, 100 μm. (**c**) The mice with the sericin-alginate IPN hydrogels embedded subcutaneously to the dorsal were imaged under the light of 470 nm with the filter allowing the passage of the emission wavelength 535 nm (left) and the light of 630 nm with the filter allowing the passage of the emission wavelength 700 nm (right).

We previously reported that the pure sericin hydrogel exhibits the photoluminescence that can be used for *in vivo* tracking^13^. To evaluate the photoluminescent property of the sericin-alginate IPN hydrogels, we used fluorescence microscopy and a small animal imaging system. The lyophilized IPN hydrogels produced fluorescence under the excitation lights that are often utilized to excite commonly used blue, green, and red fluoresent proteins (Fig. 5a). With the aid of the photoluminescence, the double networks within the IPNs could be visually differentiated (Fig. 5b). Moreover, these sericin-alginate hydrogels when subcutaneously implanted *in vivo* were readily detected and imaged using a small animal imaging system (Fig. 5c), suggesting that the fluorescence emitted from the IPN hydrogels is sufficiently strong for effective transdermal transmission. Together, these results indicate that the sericin network within the IPN hydrogels can inherit the sericin’s photoluminescent property in the presence of the ionically-crosslinked alginate network.

### The sustained drug release from the IPNs

**Figure 6:**
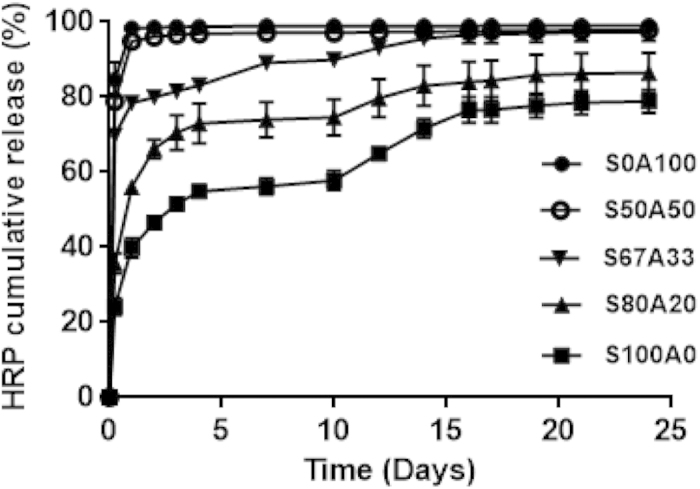
The sustained HRP release kinetics from the hydrogels. The accumulative HRP release from the pure alginate hydrogel (S0A100), the three sericin-alginate IPN hydrogels (S50A50, S67A33 and S80A20), and the pure sericin hydrogel (S100A0) was quantified over 24 days (n = 3 per group per time point).

One of the most valuable applications of hydrogels is their use as localized drug depots^38^. We next tested the drug release kinetics of these IPN hydrogels. Horseradish peroxidase (HRP) was employed as a model drug^39^,^40^. The release rates of HRP were negatively correlated with the amount of sericin within the IPN hydrogels (Fig. 6). Specifically, HRP was rapidly released from the pure alginate hydrogel up to 98.5% during the first 24 hours, whereas the release rates from S50A50, S67A33 and S80A20 were reduced to 94.8, 78.4, 56.1%, respectively. These results indicate that the sustained drug release capability can be enhanced by increasing the fractions of sericin within the IPNs, providing a convenient approach to regulate drug release rates. Although diffusion and degradation are suggested to be the main driving forces for drug release from polymeric matrices^41^, in our case the degradation dynamics of the IPNs more likely plays an important role in determining the HRP release rates as the releasing kinetics of these IPNs was similar to their degradation dynamics. This observation also suggests that electrostatic interactions between sericin protein/alginate and HRP, another factor that is thought to influence protein drug release, if involved, might not play a role as significant as degradation.

### The IPN hydrogel supports effective cell adhesion, proliferation, long-term survival, and migration

**Figure 7:**
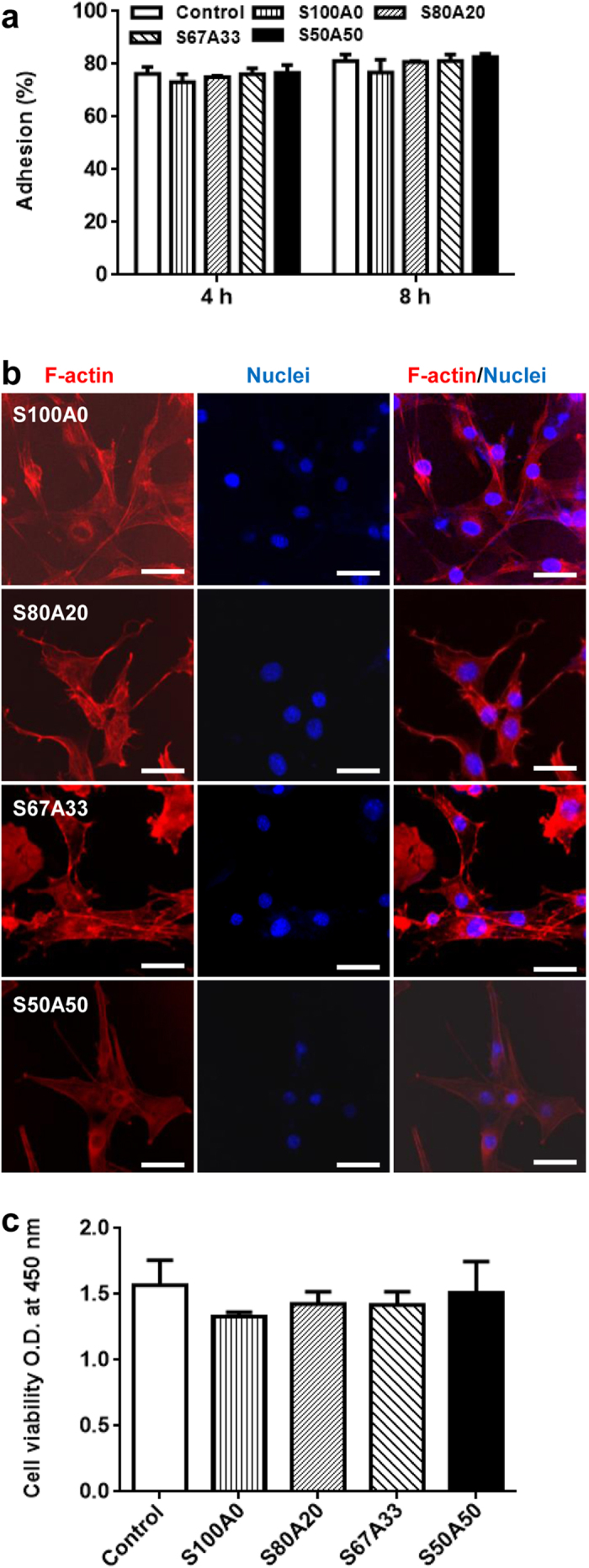
Adhesion, morphology and proliferation of C2C12 cells on the sericin-alginate IPN hydrogels. (a) The adhesion of C2C12 cells on the surfaces of the culture dishes and the IPN hydrogels (n = 3 per group). (**b**) The cells growing on the IPN hydrogels after 2-day culture were stained with rhodamine-phalloidin for F-actins (red) and DAPI for nuclei (blue). The fluorescence of rhodamine and DAPI was more intense than the background fluorescence from the IPN hydrogels, thus providing the sufficient contrast for imaging. Scale bars, 50 μm. (**c**) The cell viability on the cell culture dishes and the sericin-alginate IPN hydrogels after 3-day culture (n = 6 per group).

**Figure 8:**
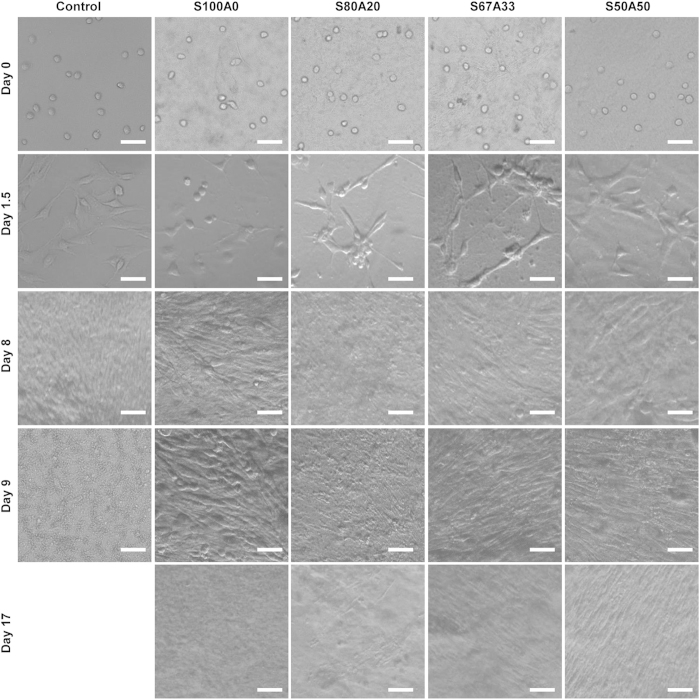
The sericin-alginate IPN hydrogels support effective cell proliferation and long-term survival. C2C12 cells were seeded at the density of 3,000 cells/ cm^2^ onto the 35-mm culture dishes (control) and the IPN hydrogels formed on the culture dishes. The cells survived and proliferated over 17 days. Scale bars, 50 μm.

**Figure 9:**
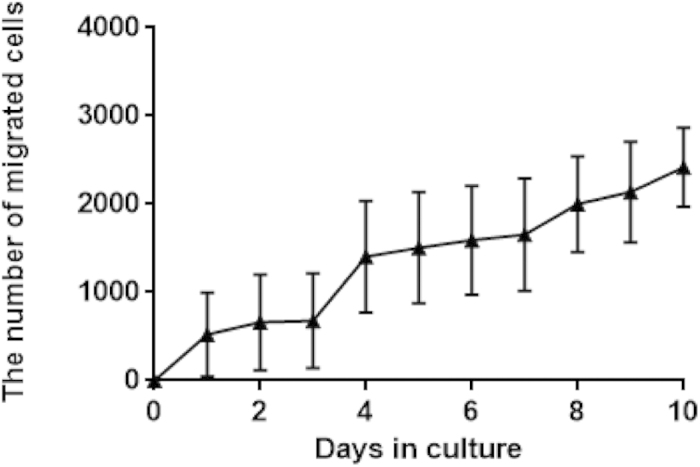
The migration of C2C12 cells from the IPN hydrogel (S80A20). The C2C12 cells were seeded onto the surface of the IPN hydrogel S80A20 (diameter 21.4 mm; thickness 2.2 mm) at the density 27,000/ cm^2^ in a 12-well plate. After 24 hours the cell-seeded hydrogels were moved to a 6-well cell culture plate to allow cells to migrate. The hydrogels were transferred to a new 6-well plate daily. After transferring the cells remaining on the plate were the cells that migrated out from the hydrogel. The number of cells were then counted (n = 3 per time point).

While alginate is known to be inefficient in promoting cell adhesion^42^, sericin is naturally cell adhesive^13^. We next examined cell adhesion on the surfaces of the series of the sericin-alginate IPN hydrogels. Regardless of the fractions of the sericin network within the IPN hydrogels, the adhesion of mouse myoblasts (C2C12) to the surfaces of the IPN hydrogels after 4- and 8-hour culture was similar to that on the pure sericin hydrogel (Fig. 7a), indicating that the involvement of sericin in the IPN hydrogels makes the surfaces adhesive. Consistently, F-actin cytoskeleton that plays a critical role in cell adhesion and migration was effectively formed in the cells cultured on the IPNs, similar to the cells growing on the pure sericin hydrogels (Fig. 7b). Furthermore, compared to the control cells cultured on the surface of the culture dishes, no significant differences in cell viability were detected between the cells cultured on the surface of the pure sericin hydrogel and those growing on the surfaces of the IPN hydrogels (Fig. 7c), suggesting that the sericin-alginate IPN hydrogels effectively support cell proliferation. We next examined the long-term survival of mouse myoblasts (C2C12) on the IPNs as long-term survival is critical for generating functional bioengineered tissues^43^. When the cells were seeded at the initial density 3,000/ cm^2^ in a 35-mm culture dish, the IPNs supported cell growth for over 17 days (Fig. 8). Since cell migration is essential for many repairing processes, such as wound healing, inflammation and tissue development^44^, we then evaluated the migration behavior of C2C12 cells in the IPN hydrogel S80A20, which had the slowest degradation rates among these IPNs. The cells continuously migrated out from the IPNs for at least 10 days (Fig. 9), indicating that the IPN’s cell-adhesive property does not interfere cell migration ability from the hydrogel, suggesting that this IPN may serve as a platform to deliver cells *in vivo*. Together, these results reveal a crucial role of the sericin network within the IPN hydrogels in promoting cell adhesion, and supporting proliferation, survival and migration.

## Conclusion

To improve the properties of alginate hydrogels, we have successfully generated an interpenetrating hydrogel containing the sericin crosslinked network and the ionically-crosslinked alginate network. With the existence of the sericin network, the IPN hydrogels exhibit many advantages in comparison to the pure alginate hydrogel, in particlular in mechanical property, degradation, photoluminescent property and cell adhesion. Moreover, the IPN hydrogel supports effective cell growth, long-term survival, and migration. With controlled bioactive molecule release property, the sericin-alginate IPNs may serve as a valuable platform for delivering cells and drugs for various tissue renergation. This work provides a rationale for the future exploration of sericin’s potential as a building block with other polymers to obtain novel IPNs with improved properties.

## Methods

### Materials

Silkworm cocoons (*Bombyx mori*, *185 Nd-s*) were obtained from Sericultural Research Institute, China Academy of Agricultural Sciences (Zhenjiang, Jiangsu, China). LF 20/40 alginate (high molecular weight, HMW) was obtained from FMC Biopolymer (Philadelphia, PA, USA). Low molecular weight (LMW) alginate was generated from HMW alginate using gamma irradiation at 50 KGy for 4 hours with a cobalt-60 source. Glutaraldehyde and CaCl_2_ were purchased from Sinopharm Chemical Reagent Co., Ltd (China). C57BL/6J mice were supplied by Department of Experimental Animals, Tongji Medical College, Huazhong University of Science & Technology (Wuhan, China). The mouse myoblast cell line (C2C12) was purchased from the American Type Culture Collection (Rockville, MD, USA).

### Preparation of the sericin-alginate IPN hydrogels

Sericin was isolated from cocoons of *185 Nd-s* silkworm that produce silk containing only sericin using LiBr method as previously reported^13^. Alginate solution was prepared by dissolving solid alginate containing equal weight HMW and LMW alginate in ultra-pure water, and stirred overnight at 4 °C. IPNs were prepared by mixing the following solutions: alginate solution (2% (w/v)), glutaraldehyde solution (25% (w/v)), sericin solution (4% (w/v)) and CaCl_2_ solution (20% (w/v)). For mixing, the volume ratio of sericin solution to glutaraldehyde solution was kept constant at 100:2 (v/v) and the volume ratio of alginate solution to CaCl_2_ solution was kept at 100:5 (v/v). According to the feed ratios (volume) of sericin solution (S) to alginate solution (A) at 1:0, 4:1, 2:1, 1:1, and 0:1, the resulting IPN hydrogels were termed, S100A0, S80A20, S67A33, S50A50, and S0A100. After mixing for 1 hour, the gel was formed within the syringe and was taken out by physically removing the syringe wall. The gel was cut into various shapes depending on experimental requirements.

### Scanning electron microscopy and porosity analyses

The morphology of the freeze-dried sericin-alginate hydrogel scaffolds was observed using scanning electron microscopy (SEM) (JSM-5610LV, Japan) with the working voltage 25 kV. All specimens were sputter coated with gold particles. 25 random pores were averaged to obtain the mean pore size for each sample using the Image Pro Plus (version 6.0.0.260). The liquid displacement method was used to determine the porosity of the sericin-alginate hydrogels. The lyophilized sericin-alginates were immersed in a known volume (*V1*) of water in a graduated cylinder for approximately 1 hour. The total volume (including water and the scaffold) was recorded as *V2*. The water-impregnated sample was then removed from the cylinder and the residual water volume was recorded as *V3*. The porosity of the scaffold (ε) was obtained by:


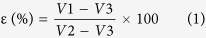


### Mechanical analysis and fourier transformation infrared spectroscopy

Cylindrical samples of the sericin-alginate IPN hydrogels (12 mm in diameter and 8 mm in thickness) were used to investigate their compression modulus on a universal testing machine (Instron 5848 MicroTester, USA) at a constant compression rate of 1 mm/ min. The analysis on the secondary structure of the sericin-alginate hydrogels was carried out on freeze-dried samples using fourier transformation infrared spectroscopy (Nexus, Thermal Nicolet, USA) in the spectral region of 4000-400 cm^−1^ with a ZnSe ATR cell.

### Quantification of equilibrium water content (EWC)

A gravimetric method was employed to evaluate the EWC of the IPN hydrogels. The freeze-dried sericin-alginate hydrogel scaffolds were allowed to equilibrate in the phosphate buffer solution (PBS) with different pH values (pH 3, pH 7.4, and pH 11) at 37 ^o^C for 24 hours. These samples were taken out and weighed immediately after the liquid on the surface was removed. The EWC of the scaffolds was calculated using the equation:


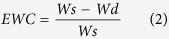


where *Wd* is the dry weight of the sample and *Ws* is the swollen weight of the sample.

### Degradation of the sericin-alginate IPN hydrogels

The specimens were immersed at 37 °C in PBS at pH 3, pH 7.4, and pH 11. The PBS was replaced daily. At pre-determined time intervals, the samples were taken out, dried and weighed.

### Photoluminescent property analyses

Lyophilized samples were observed using a fluorescence microscope (Olympus IX71, Japan) obtained under the light with different wavelengths via the software cellsens standard 1.7 (Olympus, Japan) or NIS-Elements AR 3.2 64-bit (Nikon, Japan). For *in vivo* real-time tracking analysis, the hydrogels were embedded subcutaneously in C57BL/6J mice at the age of 6 weeks. A small animal imaging device (*in-vivo* Fox pro, Bruker, USA) was used to detect the hydrogels *in vivo*.

### Horseradish peroxidase (HRP) release from the IPN hydrogels

HRP *in vitro* release from the IPNs was examined as we previously reported^13^. HRP was loaded into the IPNs by blending 1/300 (v/v) HRP (0.5 mg/ml) with the IPN gelling solution. The hydrogels (600 mg) were immersed in PBS (pH 7.4, 3 ml) at 37 ^o^C. At the pre-designed time points, the concentration of HRP in PBS was determined using ELISA.

### Cell culture on culture dishes and IPN hydrogels

Mouse myoblasts (C2C12) were cultured with high glucose DMEM media containing 10% fetal bovine serum (FBS), 100 units/ ml penicillin and 100 μg/ ml streptomycin. Prior to cell seeding, the hydrogels were immersed in fresh PBS (pH 7.4) for 3 hours and were washed by sterilized PBS three times. Next, the hydrogels were sterilized using 75% ethanol for 1 hour. The cells in DMEM were seeded in a dropwise manner at the density of 3,000 cells/ cm^2^ onto the hydrogels cast in 35-mm culture dishes. All cells were cultured using complete cell culture media at 37 ^o^C in 95% oxygenated tissue culture hood. The culture medium was replaced daily.

### Cell adhesion and viability analysis

To investigate the cell adhesion property of IPN hydrogels, sericin-alginate IPN hydrogels were generated at the well bottom of a 12-well cell culture plate using 1000 μl reaction buffer containing sericin, alginate, glutaraldehyde, and CaCl_2_. Prior to cell seeding, the IPN hydrogels were washed using sterilized PBS three times followed by an immersion in 75% ethanol for 1 hour. Then, the hydrogels were washed by PBS three times and kept in DMEM for 1 hour. C2C12 cells were seeded at the density of 500,000/ well onto the hydrogels cast in 12-well plates or cell culture dishes (as controls) and incubated at 37 °C as described above. After 4 hours and 8 hours the hydrogels were taken out carefully and washed gently with PBS (pH 7.4). By subtracting the number of cells washed out by PBS, the number of cells adhering to each hydrogel was calculated. CCK-8 assay was used to assess cell viability at Day 3 after cells were loaded at the density of 3,000/ well onto the sericin-alginate IPN hydrogels cast in the wells of 96-well plates.

### F-actin confocal imaging analyses on cells growing on the IPN hydrogels

C2C12 cells were seeded at the density of 3,000 cells/ cm^2^ onto the 35-mm culture dishes (control) and the IPN hydrogels formed on the culture dishes for 2-day culture. The IPN hydrogels with cells were submerged for 10 minutes in PBS (pH 7.4) that was then replaced with 4% paraformaldehyde. The fixed samples were stained with rhodamine-phalloidin and 4′, 6-diamidino-2-phenylindole (DAPI), and then were examined by a confocal laser-scanning microscope (Nikon A1Si, Japan).

### Cell migration analysis

The sericin-alginate IPN hydrogels (S80A20) were generated at the well bottom of a 12-well cell culture plate using 1000 μl reaction buffer containing sericin, alginate, glutaraldehyde, and CaCl_2._ Prior to cell seeding, the cast IPN hydrogels were washed using sterilized PBS three times followed by a 1-hour immersion in 75% ethanol. Then, the hydrogels were washed by PBS three times and kept in DMEM for 1 hour. After the formation of these hydrogels, 1 × 10^5^ cells suspended in 500 μl DMEM were loaded onto the hydrogels. The plates were then incubated at 37 ^o^C for 24 hours. Using the migration assay previously reported^45^, the hydrogels with the cell-seeded side up were transferred to a new 6-well plate to allow cells to migrate. The 6-well plates were replaced daily. After transferring, the cells in the plates were collected and counted.

### Statistical analysis

All data were expressed as mean ± SD. Each experiment was performed at least three times. The CCK-8 assay was run in 6 replicates per test sample. Data were analyzed by ANOVA tests. *P* ≤ 0.05 was considered statistically significant and *P* ≤ 0.01 was highly significant.

## Additional Information

**How to cite this article**: Zhang, Y. *et al.* Design and performance of a sericin-alginate interpenetrating network hydrogel for cell and drug delivery. *Sci. Rep.* **5**, 12374; doi: 10.1038/srep12374 (2015).

## Figures and Tables

**Table 1:** Pore size and porosity of the sericin-alginate IPN hydrogels frozen at −80 °C.

Sericin-alginate hydrogels	S100A0	S80A20	S67A33	S50A50
Average pore size (μm)	138.66 ± 67.07	105.23 ± 75.86	98.57 ± 47.45	79.82 ± 37.88
Porosity (%)	91.69 ± 0.05	90.44 ± 0.08	91.78 ± 0.34	92.51 ± 0.10

Data are shown as mean ± SD (25 random pores per sample, 3 samples for each analysis).
